# Medical importance of candiru catfishes in Brazil: A brief essay

**DOI:** 10.1590/0037-8682-0540-2020

**Published:** 2021-03-22

**Authors:** Vidal Haddad, Jansen Zuanon, Ivan Sazima

**Affiliations:** 1 Universidade Estadual Paulista, Faculdade de Medicina de Botucatu, Departamento de Infectologia, Dermatologia, Radioterapia e Diagnóstico por Imagens, Botucatu, SP. Brasil.; 2 Instituto Nacional de Pesquisas da Amazônia, Manaus, AM, Brasil.; 3 Universidade Estadual de Campinas, Museu de História Natural, Campinas, SP, Brasil.

**Dear Editor,**

The type of catfish referred to as candirus, is a member of the Trichomycteridae and Cetopsidae families^1^ ^,^ ^2^. The subfamilies Vandelliinae and Stegophilinae (Trichomycteridae) and Cetopsinae (Cetopsidae) are relevant to human medicine, including forensics^1^ ^,^ ^2^ ^,^ ^3^ ^,^ ^4^. Herein, we present a brief essay on candirus and their relationship with human health and death. We examined published accounts and used our personal observations on the subject.

Candirus of the genus *Vandellia* are small (usually around 4-8 cm but can reach a size of 20 cm), have an elongated body (Figure 1A), and their mouths contain sharp, needle-like teeth. The two most extensively studied *Vandellia* species feed on blood from the gill arteries of larger fish (Figure 1B). These candirus may remain within the gill chamber of the fish host for up to 145 seg^5^. There is no robust evidence that vandelliine candirus react to ammonia or blood in water^6^, but they allegedly enter the human urethra or other natural orifices to feed on blood^7^ ^,^ ^8^ ^,^ ^9^. However, such cases are disputed due to the apparent lack of solid evidence^9^.

**FIGURE 1:**
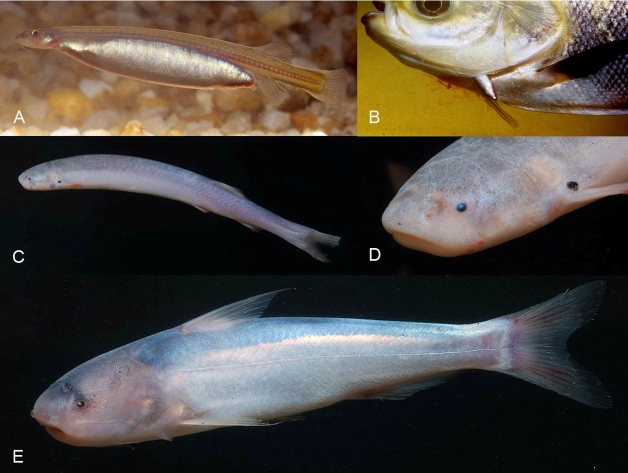
**(A)**: The blood-feeding *Vandellia cirrhosa* from the subfamily Vandelliinae after a full meal of blood and **(B)**: while taking blood from the gills of a fish host. Note the engorged belly of the candiru after a blood meal, and blood leaking from the gill opening of the fish host. **(C)**: The carrion-eating candiru *Pareiodon microps* from the family Stegophilinae. **(D)**: A close-up of the head. Note the shape of the mouth and compare it with that of the whale candiru. (**E**): The whale candiru *Cetopsis coecutiens* from the subfamily Cetopsinae. Photos by Ivan Sazima and Jansen Zuanon (A, B) and by Jansen Zuanon and Efrem J. G. Ferreira. (C, D, E).

However, cases of attacks on humans by a vandelliine candiru that inflicts wounds on the body of the victim have emerged recently. This candiru was identified to be a part of a scientifically undescribed genus and species^2^ and is referred to here as the human-biting candiru. This candiru fastens itself to the victim’s body with its specialized teeth (and perhaps using interopercular spines) and feeds on blood (Figure 2A). The fish is difficult to remove from the victim (Figures 2B and D) because of the forceful bite exerted by the powerful head muscles. 

Upon forceful removal of the fish, the wound bleeds for a while (Figure 2D ). The lesion caused by human-biting candiru is elliptical, similar to the wound inflicted by the blood-feeding *Vandellia cirrhosa* on the large arteries of its fish hosts^5^.

**FIGURE 2:**
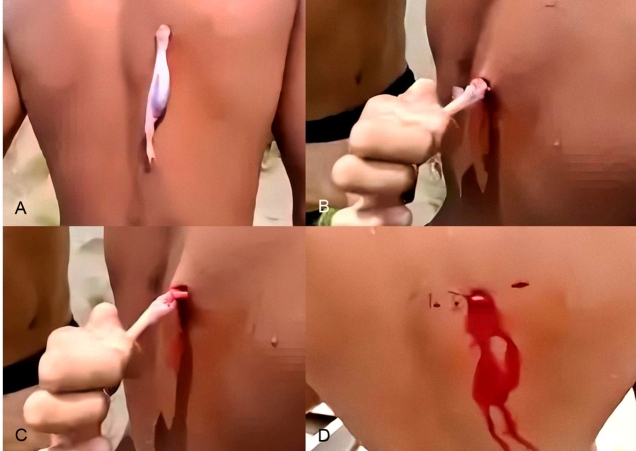
**(A)**: The human-biting candiru from the subfamily Vandelliinae, a part of a scientifically undescribed genus and species, fastened to the back of a boy. **(B-C)** Upon a forceful removal, the fish’s mouth leaves a **(D)** bleeding elliptical lesion at the attachment point. Note the candiru’s abdomen full of blood, its strong hold on the victim, and another bite to the right of the bleeding one. Video stills by Kalebe Pinto.

Candirus of the subfamily Stegophilinae are small-sized fishes (about 10-15 cm) that semi-parasitize large fishes^10^. They fasten to their victim with the disproportionally expansible sucking disc-like mouth, bite, and spread the opercular and inter-opercular spines into the wound to remain attached, scraping off mucus or scales^10^ ^,^ ^11^ or taking blood^12^. Within the Stegophilinae subfamily, the candiru *Pareiodon microps* stands out as a species of forensic importance due to its carrion-eating habits^2^ ^,^ ^11^. This candiru is slender and long (Figure 1C) and is unlikely to be confused with the stouter carrion-eating whale candirus, despite the shape of its mouth (Figure 1D). This stegophiline candiru joins the whale candirus while feeding on dead vertebrates^2^ ^,^ ^13^, which may include humans.

The whale candirus of the subfamily Cetopsinae are unrelated to vandelliine candirus^1^. Cetopsine candirus are stockier and larger (up to about 30 cm) than vandelliine and stegophiline candirus (Figure 1E). Only two of about 35 species of whale candirus are carrion-eaters that tear off chunks of tissue with their specialized, pointed, razor-sharp teeth on the mandible^1^ ^,^ ^14^ ^,^ ^15^. These fishes penetrate the bodies of drowned or otherwise dead animals, including humans, and feed on viscera and musculature^1^ ^,^ ^15^. Whale candirus may attack live fish in gillnets and occasionally, humans^1^ ^,^ ^7^. Human corpses attacked by whale candirus have round deep holes on the body surface, which correspond to exit or entry holes^1^ ^,^ ^15^.

The two carrion-eating whale candirus display different behaviors when feeding^1^. *Cetopsis candiru* bites and makes a rotational movement along its vertical axis, tearing off an almost round piece of tissue and tunnels its way into the corpse^7^. Then, it proceeds to enter the body cavity, eating the corpse from the inside out^1^ ^,^ ^15^. 

This candiru species display a feeding frenzy that may involve tens to hundreds of individuals^1^ ^,^ ^7^. However, the whale candiru *Ceptosis coecutiens* bites quickly and tears off chunks of tissue and then withdraws to attack again^1^. Attacks by these two *Cetopsis* species cause deep lesions on human corpses, and the bite of the whale candirus is so strong that it leaves circular marks even on the skull bone of the victims^15^.

Feeding frenzies of *C. candiru* on dead vertebrates may be joined by the carrion-eating catfish piracatinga, also known as *Calophysus macropterus*, of the family Pimelodidae^7^ ^,^ ^13^. This long-whiskered catfish also has razor-sharp teeth, appropriate to tear pieces of flesh off dead bodies, including those of humans^15^.

Finally, it is important to remember that reports on candirus and their relationship to human health and death are biased by imprecision, second- and third-hand accounts, misconceptions, and folk tales^1^ ^,^ ^2^ ^,^ ^7^ ^,^ ^8^ ^,^ ^9^. Due to the above-mentioned biases, several accounts of the impact of candirus on people are dubious or at least imprecise, even in some scientific reports and books.

## References

[1] de Pinna, Queiroz LJ, Torrente-Vilara G, Ohara VM, Pires THS, Zuanon J, Doria CRC (2013). Peixes do Rio Madeira.

[2] de Pinna MCC, Queiroz LJ, Torrente-Vilara G, Ohara VM, Pires THS, Zuanon J, Doria CRC (2013). Trichomycteridae. Peixes do Rio Madeira.

[3] Lesimann W, Queiroz T, Camargo LMA (2020). Child injured by suspected catfish (Cetopsis sp.) bite in river, Humaitá, Amazonas, Brazil. Rev Soc Bras Med Trop.

[4] Valente-Aguiar MS, Falcão AC, Magalhães T, Dinis-Oliveira RJ (2020). Cadaveric ichthyofauna of the Madeira River in the Amazon basin: the myth of man-eating piranhas. Forensic Sci Med Pathol.

[5] Zuanon J, Sazima I (2004). Vampire catfishes seek the aorta not the jugular: candirus of the genus Vandellia (Trichomycteridae) feed on major gill arteries of host fishes. Aqua J Ichthyol Aq Biol.

[6] Spotte S, Petry P, Zuanon J (2001). Experiments on the feeding behavior of the hematophagous candiru Vandellia cf. plazaii. Env Biol Fish.

[7] Goulding M (1980). The fishes and the forest-explorations in Amazonian natural history.

[8] Spotte S (2002). Candiru-life and legend of the bloodsucking catfishes.

[9] Bauer IL (2013). Candiru-a little fish with bad habits: need travel health professionals worry? A review. J Travel Med.

[10] Baskin JN, Zaret TM, Mago-Leccia F (1980). Feeding of reportedly parasitic catfishes (Trichomycteridae and Cetopsidae) in the Río Portuguesa basin, Venezuela. Biotropica.

[11] Do Nascimiento C (2015). Morphological evidence for the monophyly of the subfamily of parasitic catfishes Stegophilinae (Siluriformes, Trichomycteridae) and phylogenetic diagnoses of its genera. Copeia.

[12] Burgess WE (1989). An Atlas of Freshwater and Marine Catfishes.

[13] Beltrão H, Porto-Braga TM, Schwartz-Bensaken Z (2017). Alternative bait usage during the piracatinga (Calophysus macropterus) fishery in the Manacapuru region, located at the lower Solimões-Amazonas River, Amazon basin, Brazil. Pan-Am J Aquat Sci.

[14] Vari RP, Jr. CJ Ferraris, de Pinna CC (2005). The Neotropical whale catfishes (Siluriformes: Cetopsidae: Cetopsinae), a revisionary study. Neotrop Ichthyol.

[15] Rocha MS, Zuanon J, Queiroz LJ, Torrente-Vilara G, Ohara VM, Pires THS, Zuanon J, Doria CRC (2013). Pimelodidade. Peixes do Rio Madeira.

